# Measuring Gastronomic Image Online

**DOI:** 10.3390/ijerph16234631

**Published:** 2019-11-21

**Authors:** Estela Marine-Roig, Berta Ferrer-Rosell, Natalia Daries, Eduard Cristobal-Fransi

**Affiliations:** Department of Business Administration, University of Lleida, Catalonia, C. Jaume II, 73, 25001 Lleida, Spain; berta.ferrer@aegern.udl.cat (B.F.-R.); ndaries@aegern.udl.cat (N.D.); ecristobal@aegern.udl.cat (E.C.-F.)

**Keywords:** culinary tourism, gastronomy, restaurant, dining, destination image, user-generated content, big data analytics, online travel review, TripAdvisor, Canary Islands

## Abstract

Culinary or gastronomic tourism has become one of the main exponents of cultural tourism and a key element of a destination’s image identity. Since travellers consult and produce online travel reviews (OTR) before and during a trip, this research aims to provide and implement a framework for analysing OTRs of dining establishments to measure their contribution to destination image formation in their designative (cognitive) and appraisive (affective and evaluative) aspects. To do this, a website was selected from which to download OTRs, extract useful information from the textual and paratextual elements, build a keyword frequency matrix, and perform a quantitative and thematic content analysis. This method was applied to a random sample of 500,000 OTRs from the TripAdvisor restaurants section, written in English, between 2013 and 2017, by tourists visiting the Canary Islands. Results show that, although the gastronomic image of the destination is positive in general, the local and regional gastronomy representative of the community’s sociocultural identity is not the most popular nor the best valued in tourists’ comments. This research shows a method to measure the main aspects that make up the gastronomic image of a destination and that allow for extracting insights and business intelligence through big data from user-generated content.

## 1. Introduction

In today’s global, competitive, and dynamic environment, many destinations have similar characteristics and therefore have a growing need to differentiate [1]. This means that, in many cases, gastronomy has become a differentiator, helping promote the image, the host community’s culture, and identity of destinations [2,3,4]. Culinary or gastronomic tourism is related to food and eating experiences of travellers. Several authors have analysed the relationship between food and tourism from the perspective of food as part of a reflection of the local culture [5,6,7,8,9]. Culinary tourism includes not only food tasting and the enjoyment of the dining experience but also has an important educational role on the destination’s culture and a great potential to contribute to sustainability [10]. Food is culture within the cultural act of tourism [4]. According to the International Culinary Tourism Association (ICTA), there is a strong positive correlation between tourists who are interested in cuisine and those who show an interest in cultural attractions and sustainable social network development, generating positive socioeconomic and environmental impacts.

The gastronomic tourist (who gives value to gastronomy as a means of socialization and exchange of experiences) is increasingly demanding and has a higher level of spending than the average tourist [11,12]. Restaurants attracting tourists have become the target of many destinations as they help extend the length of stay, increase tourism spending on local products, promoting the proximity production, the small-scale agriculture, and ultimately create greater sustainability [13]. This is why many destinations are increasingly positioned as gastronomic [14,15,16]. However, previous studies found a lack of awareness of regional products and confusion regarding national dishes in some destinations, which may have negative impacts at the socio-cultural level [9].

In spite of this, the current gastronomic tourist is a much more informed customer and, thanks to new technologies, s/he often plans and books their holidays and leisure time based on experience, online reviews (OTR), and recommendations from other customers transmitted through electronic word-of-mouth (eWoM) marketing [17]. For instance, focusing on American tourists, [18] surveyed 2025 leisure travellers with the question “In the past 12 months, which of these internet technologies or services have you used to help plan your leisure travel? (Select all that apply)” (p.5). Results revealed that 58.2% of respondents used any user-generated content (UGC) source (OTRs: hotels 32.5%, destinations 29.6%, and restaurants or activities 30.8%). In addition, travel experiences based on gastronomy and food help build loyalty [19].

In general terms, Spain received 82 million international tourists in 2017 [20], surpassing the United States for the first time and reaching the second position in terms of tourist arrivals, just behind France. International tourists made a total expenditure of 87,000 million euros, of which it is estimated that approximately 13,000 million was intended for food, and for 15% of tourists (roughly 12 million people), the main reason to visit Spain was gastronomy. According to the extrapolated data of the tourist expenditure survey [21], in the first quarter of 2018, the Canary Islands were the first main destination of foreign tourists arriving in Spain. In terms of the level of expenditures made, this autonomous community had the greatest weight (32.2% of the total), followed by Catalonia (20.2%).

Perceived cognitive and emotional images affect satisfaction [22], and the affective image has a significant effect on loyalty [23]. Thus, it is important to analyse tourists’ opinions on the destination’s resources in order to improve its image, position, tourist loyalty, direct marketing efforts, strategies, planning, and management. However, scientific literature based on the analysis of OTRs on restaurants is scarce and ranges between 12% [24] and 14% [25] of the hospitality and tourism online review research.

This study aims to measure the gastronomic image involved in building the tourist destination image (TDI). To do this, designative (cognitive) and appraisive (evaluative and affective) components of the destination image are analysed using big data from TripAdvisor (500,000 OTRs written in English, between 2013 and 2017) on restaurants in the Canary Islands, the most-visited region of the European Union by number of overnight stays in tourist accommodations.

## 2. Gastronomic Image Online within Tourist Destination Image

**Figure 1:**
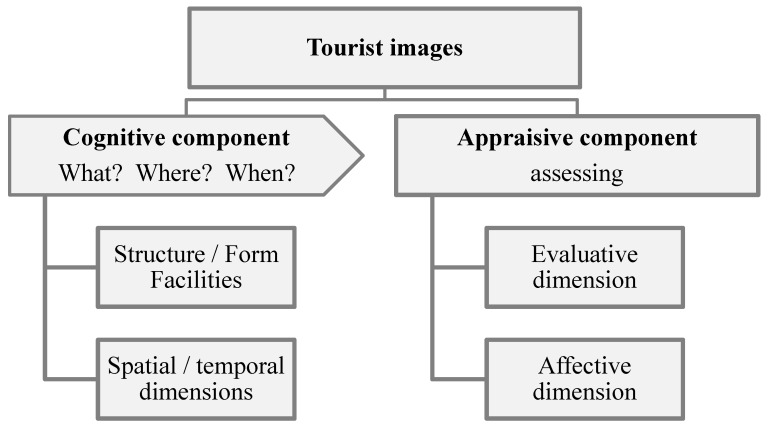
Destination image components. Source: Authors, derived from Marine-Roig [31].

The perceived image of tourist destinations is very important as it affects the behaviour and satisfaction of tourists, as well as their decision-making and future recommendations [26]. The overall image of a destination is formed by the cognitive and affective components [27,28] of the classical model [29,30]. Figure 1 shows a proposed extension of this model based on Marine-Roig [31], to incorporate spatial and evaluative dimensions foreseen by Pocock et al. [32], and the temporal dimension [33]. The cognitive component of the image is belonging or related to knowledge of tourist resources. It answers the following questions: “What is it?”, “What does it offer?”, “When is it perceived?”, and “Where is it situated?”. Structure, form, and facilities are distinguished in this component. Facilities include relatively abstract mental images such as when a structure is identified as a restaurant [34]. In contrast, the appraisive component refers to the appreciation, consideration, or evaluation of the above items and depends directly on the cognitive component (requires prior knowledge of attraction, service, or activity). Within this component, the evaluative aspect (score) and its affective aspect (relative to sensitivity, impression, or sensation caused) are distinguished.

### 2.1. Gastronomy as a Factor Promoting the Image and Identity of Destinations

Since some researchers have shown the positive influence of culinary experiences on destination image and loyalty [35,36], gastronomy is increasingly used as a hallmark in promoting destinations. Proof of this is the number of studies that analyse the relationship between gastronomy and tourism as a factor in promoting the image and identity of destinations [37]. According to Enright et al. [38], gastronomy is the second factor of attraction for visiting Hong Kong. Fox [39] suggests reinventing the gastronomic identity of Croatia to gain a competitive advantage. Getz [40] discusses the importance of wine tourism destinations, such as in the USA, Canada, Australia, New Zealand, and Europe, with recommendations for those destinations with potential to develop in this niche market. Duttagupta [41] analyses the contextual vacuum regarding the concepts of image, satisfaction, and behavioural intentions and applies these to gastronomy and travel experience in India. Ab Karim et al. [3] compare the perceived image as a culinary destination for tourists and potential business between France, Italy, and Thailand. Chang et al. [42] go further and analyse the gastronomic behaviour of Chinese tourists in a different environment, such as Australia, and how their culture influences their restaurant choices, the type of food they order, schedules, and the assessments and recommendations they make. If tourists consume local food and they enjoy, it represents as a great value for sustainable tourism and the development of destinations, that experience allows them to feel deep in the culture, history, and heritage of the place and connect with the personality and way of life of its inhabitants. If they appreciate the experience of consuming these types of products, visitors not only feel deep in the culture and identity of the area, they become ambassadors of the destination and recommend it after their trip [43].

### 2.2. Content Generated by Travellers in the Restaurant Industry

Customers are increasingly using the internet as a source of information on tourism products and services, such as hotel reservations, car rentals, restaurants, flights, and tourist packages [44,45,46]. In fact, according to a survey at the European level (Eurobarometer, 2016), the internet was the most common way to organize a trip (two out of three people use it for this purpose), and the recommendations of websites that collect OTRs were the second most important source of information after recommendations from family and friends.

Many authors argue that the UGC is highly influential and credible [47,48,49] and can have a significant effect on the behaviour, decisions, expectations, and perceptions of tourists. The influence of UGC and social media is growing every day due to the expansion and increasing use of reviews, their acceptance, trustworthiness, capacity to meet tourists’ needs and interests, and the many advantages they present for users [50]. The intentionality is different behind user-generated images online (e.g., social relationship, self-expression, social recognition, leisure) and the other agents’ (among them, restaurants) images that render user-generated images credible in the eyes of other users. The tourist’s image construct of projected vs. perceived outline must be rethought and widened to include and emphasize the concept of the image transmitted by tourists (which is, at the same time, a perceived-projected image of tourists) [51]. These studies have aroused great interest from companies and tourism organizations, as well as researchers [52].

Online reviews have become an important source of information for tourism businesses looking to improve their marketing strategies and the satisfaction of customers [53]. Given the large number of OTRs, methods for processing big data, which allow extracting useful information from user reviews, are necessary [31].

The widespread use of social networks and the increased use of online reviews are changing the way restaurants obtain data on the behaviour and preferences of their guests [54]. In the past, hoteliers and restaurateurs got this valuable information from exhaustive surveys after a guest’s stay or through “mystery shopping” tools, which assessed the level of service provided, areas for improvement, and customer satisfaction.

Nowadays, gastronomy has become a relevant category due to the number of related terms and concepts that appear specifically in the user-generated content by visitors, and several studies focusing on the effects of the reviews in the restaurant industry have been conducted. For example, Kim et al. [55] analyse the influence of the reviews of social networks in the financial performance of restaurants; Luca [56] studies the effects of reviews on the demand for restaurants and the financial results. Zhang et al. [57] compare the reviews generated by users based on their experience and those written by professionals and analyse the degree of influence on the behaviour of consumers when deciding to visit a restaurant, recognizing that reviews created by consumers and professional editors often do not differ in their content. However, users’ reviewers are perceived as much more credible. Salehi-Esfahani et al. [58] realize how extreme opinions online (very positive or very negative) and the credibility of the person posting them significantly and positively affect the usefulness of restaurant customers. Gkoumas [59] indicates that elements such as affinity and age of the person sharing the review, context, content and the response of the owner increased the empathy of the visitors for particular restaurants. Tiago et al. [60] conclude that the most important variable in restaurants’ UGC is the quality of the food, and that food is much more than just its taste.

Other studies focus on the analysis of the territorial specialisation of the image based on UGC in travel blogs and reviews, determining that food and wine are an essential part of the image identity and differentiation between territorial brands [61,62]. In a broad sense, the destination image identity includes different values, elements, ideas, experiences, and feelings that are transmitted within an image and represent and define that destination [63]. In this respect, restaurants are one of the central elements for the development of culinary tourism in destinations and the transmission of destination image and identity through this form of cultural tourism. However, no studies have been found that analyse online travel reviews to unveil the gastronomic image identity of a destination.

## 3. Materials and Methods

To analyse the influence that the opinions of eating establishment customers have on the image of Spanish tourist destinations, the Canary Islands (the region with the most tourist influx in Spain) were chosen as the tourist destination for this case study. The method consisted of selecting the most suitable travel-related websites, downloading the OTRs, processing the data, and carrying out the content analysis based on the categorisation from the most frequent keyword terms matrix [26].

### 3.1. Case Study: Canary Islands

Europe is the region with the highest tourist influx worldwide, with levels double that of the next classified region [64]. The European Commission annually publishes a ranking of the most tourist-related European regions by the number of overnight stays in tourist accommodation [65]; it ranks the Canary Islands in the first place, with more than 100 million annual overnight stays.

**Figure 2:**
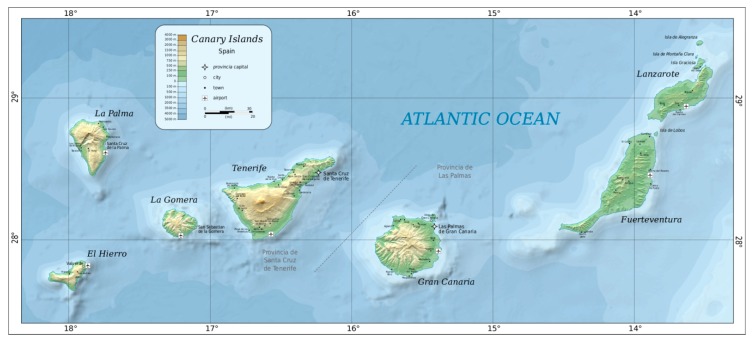
Map of the Canary Islands. Author: Oona Räisänen (Mysid) [Public Domain].

The Canary Islands are an archipelago in the Atlantic Ocean, one of the 17 regions that are part of Spain. They are also one of the outermost regions of the European Union. The archipelago comprises seven islands: El Hierro, La Gomera, La Palma, Tenerife, Fuerteventura, Gran Canaria, and Lanzarote (see Figure 2). The Canary Islands have a population of 2,167,685 inhabitants (2018), making it the eighth largest Spanish region by population. This population is concentrated mainly on two islands: Tenerife and Gran Canaria. Due to their location, the Canary Islands have a subtropical climate. Their natural attractions, beaches, and landscape make the islands a very attractive tourist destination. Average temperatures in the archipelago are between 22° in summer and 19° in winter, allowing for non-seasonal tourism.

Table 1 shows different data on the Canary Islands as a tourist destination, such as the number of tourists visiting the islands, the average expenditure, the number of restaurants, and the cost of restaurant activity (from 2013 to 2017).

Table 2 shows where tourists in the Canary Islands eat and buy their meals. Some pay full board (3–5%) or half board (18–20%) at hotels, others pay for meals through their travel agency, and about 50–75% pay the restaurants directly. However, for example, tourists in half-board hotels usually eat lunch outside in restaurants, and tourists on all-inclusive trips also eat outside in restaurants during their trip. Others have paid meals directly in the destination’s restaurants. Therefore, about 95% of tourists to the Canary Islands eat meals in restaurants outside hotels.

The development of local cuisine based on local products as a tourist resource helps the sustainability and identity of the territory, increasing the level of local production and distribution and ensuring respect for tradition and heritage. The archipelago is also known as a ’miniature continent’ due to its variety of landscapes and rich and diverse gastronomic offerings. The archipelago offers the Spanish gastronomic culture, with Latin American treats and African influence, with high-quality products of the territory, native fish, pork, rabbit, and goat combined with potatoes of protected designation and origin high-quality wines and cheeses; however, nowadays, only 10% of restaurants of the destination make use of local production.

### 3.2. Data Collection

Massive data processing requires information structured to automate its collection, debugging, arrangement, and analysis. In the field of hospitality and tourism, there is a huge volume of semi-structured UGC on platforms dedicated to the online booking of trips, hotels, restaurants, and other tourism-related activities. Applying a weighted formula [66] based on variables like visibility (quantity and quality of inbound links), popularity (visits received and web traffic in general), and size (number of entries related to the case study), a ranking of websites hosting travel blogs and/or OTRs was built.

TripAdvisor appears in the first position as the most suitable website for the case study. This selection matches that of most authors who investigate hospitality and tourism through online reviews [25,67]. TripAdvisor has basically three sections that host many OTRs: things to do, hotels, and restaurants. In these three sections, there may be comments or opinions about food and wine, but the vast majority of opinions and ratings related to gastronomy are concentrated in the restaurants section. As seen in Table 1, the majority of visitors to the Canary Islands eat in restaurants outside hotels, and thus, the restaurant section of TripAdvisor can be a good data source to analyse the gastronomic image online. Moreover, this gastronomic image online has the value that if people search for opinions on the destination’s gastronomy, they will encounter these restaurant experiences through search engines [68]. TripAdvisor has more than three million opinions on the Canary Islands, of which over half a million are written in English and refer to restaurants. Using a web copying programme with appropriate filters [26], we proceeded to download the OTRs hosted in the restaurants section of TripAdvisor [69].

**Figure 3:**
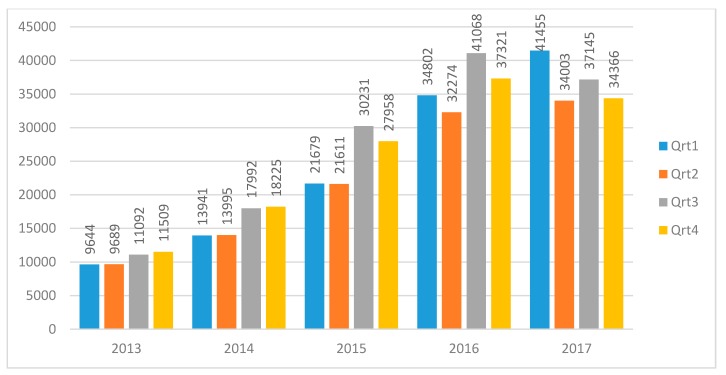
Restaurant reviews by year and quarter. Source: sample of 500,000 TripAdvisor online travel reviews (OTRs).

Once all the OTRs were written in English (619,149) and downloaded, the OTRs published between 2013 and 2017 (539,124) were selected. Subsequently, defective OTRs were eliminated (e.g., untitled or titled reviews composed of figures and other non-alphabetic characters). In order to provide readers with an easily visible number, a set of 500,000 OTRs was selected by generating for each OTR a random number between zero and one, with 15 decimal places. Once the dataset was ordered by that numbering, the OTRs after position 500,000 were removed. This random sample of 500,000 of the total OTRs for restaurants, posted in English between 2013 and 2017 (Table 3 and Figure 3) represents the vast majority of opinions regarding the case study. The English language was selected because it is the most representative of tourists who write reviews about attractions, activities, or services in the Canary Islands.

Table 3 shows the restaurant reviews of the Canary Islands by island and year. The first year included in the study (2013) represents only 8.39% of the total reviews analysed, while reviews posted in 2016 and 2017 represent more than 29% each. The number of reviews increased year by year, from almost 42,000 reviews in 2013 to almost 147,000 in 2017. Regarding island-specific reviews, the most prominent islands are Tenerife (37.12% out of total reviews included in the analysis) and Lanzarote (31.33%). Data presented in Figure 3 go beyond and include temporality. In general, the first and third quarters collect a higher number of reviews.

### 3.3. Data Mining

Data mining is the process of extracting information from a large dataset and structures it to facilitate the discovery of patterns. Web pages downloaded from TripAdvisor in plain text (no multimedia files or other attachments) were between 400 and 500 Kb in size. The information useful for the case study was less than 5% of the content of the page [26]. Useful data was extracted using a text search utility that supported expressions (regex) of regular language (search patterns). The paratextual elements [70] and HTML (HyperText Markup Language) metadata of OTR web pages [68] provided the data necessary to measure the perceived image, placing it in space and time (title, language, date, location, type or theme, rating, etc.).

For example, searching for <title> [71], the programme extracted from each file the title of the web page consisting of the start tag <title>, the title of the OTR written by the reviewer, plus the information added by the webmaster (name and location of the resource) and end tag </title>. In order to process this information, the data were cleaned and separated by semicolons (;) with regex implemented by the search and replace utility and stored in CSV files (comma separate values), which are plain text compatible with any text editor, spreadsheet, or database.

### 3.4. Content Analysis

The content analysis was based on techniques for mapping symbolic data in a matrix of data suitable for statistical analysis [72]. A common technique is quantitative analysis for the frequency of words and their categorization. It is assumed that the words most frequently mentioned are those that reflect the greatest interest [73]. Categories are groups of words with meaning and/or a similar connotation. The categories should be mutually exclusive and exhaustive for a rigorous analysis. To analyse the image perceived by customers of dining establishments, cognitive and appraisive components of the overall image (Figure 1) were considered.

#### 3.4.1. Cognitive Component

What a gastronomic establishment is and what it offers must be determined in order to classify it into a category. In the TripAdvisor restaurants section, cafes, bars, and any other establishment that offers the consumption of some type of food were included. The establishments sponsored by TripAdvisor allow multiple classifications, such as region (e.g., American, Asian, European), country (e.g., Chinese, Spanish, Indian, Italian, Japanese, Mexican), and type of food (e.g., seafood, steakhouse, vegetarian, vegan), eminently related to the cultural identity of the gastronomic experience. The Canary Islands are located in the Atlantic Ocean, close to the African coast, and their cuisine may be influenced by that of the South American countries. However, it is a European region populated mostly by Spanish citizens. That is why many restaurant owners, when they register on TripAdvisor, classify their establishment as specialising in Spanish, European, or Mediterranean cuisine. One limitation of the TripAdvisor OTRs is that the same dining establishment may be classified by different concepts, which makes the analysis more difficult.

To analyse the spatial dimension of the image at a local community level, the archipelago was divided into eight islands (Table 3), to which an abbreviation was assigned: Tenerife (Ten), Gran Canaria (GrC), Lanzarote (Lan), Fuerteventura (Fue), La Palma (LaP), La Gomera (Gom), El Hierro (Hie), and La Graciosa (Gra). The time dimension uses the date of the review as a reference (Figure 3) because, given the wide availability of smart mobile devices, reviews are posted online almost instantly or shortly thereafter.

#### 3.4.2. Appraisive Component

In order to analyse the evaluative dimension, the rating awarded by customers of the dining establishments is available in the review. The TripAdvisor system allows a rating of one to five bubbles. To facilitate comparisons in this case study, ratings were converted to a scale of zero to ten: 5* = Excellent (10), 4* = Very good (7.5), 3* = Average (5), 2* = Poor (2.5), and 1* = Terrible (0). The weighted average score was calculated (0 to 10) for each establishment surveyed. Moreover, Excellent and Very good ratings are considered positive, Poor and Terrible considered negative, and Average considered neutral.

Regarding the affective dimension, this is about assessing the moods, sensations, or feelings the customers express in the review. Among the paratextual elements of the review, the title is paramount because it summarizes and highlights the impressions perceived by its author [70]. It is also rich in qualifying adjectives, exclamations, and recommendations, which allows classification in expressing positive, negative, or neutral feelings. For example, “amazing”, “never disappoints”, or “wow!” reflect positive impressions; conversely, “avoid”, “never again”, or “yuck!” reflect negative impressions.

#### 3.4.3. Data Processing

To generate the frequency tables, the algorithm described in Marine-Roig [31] implemented in Java was used. The algorithm needs a list of composite keywords (groups of two or more words that have a different meaning for each word) and another list of non-significant words (stop words) for the case study (determiners, pronouns, prepositions, conjunctions, and adverbs), in both Spanish and English languages. In addition, you must define the word separator characters (in this case, considered separator characters are not letters in Spanish or English). In case of overlap, the algorithm prioritizes composite keywords; for example, “not good” (two words) takes precedence over “good” (single keyword) and “not” (stop word). When two composite keywords overlap, the first that appears in the list has priority. Once a frequency table was generated, the keywords were classified according to the categories described in the previous section.

## 4. Results and Discussion

In the data mining phase, we obtained two dimensions of the perceived gastronomic image: space (Table 3) and time (Figure 3). The other dimensions were obtained through quantitative and thematic content analysis of OTRs, based on the frequency of keywords (Table 4), categorisation, and TripAdvisor ratings.

Table 4 shows keywords that appeared most frequently in restaurant OTRs. This table is significant for the affective dimension of the image, given the many adjectives that qualify feelings and moods (e.g., amazing, excellent, delicious). Other common words that appear in restaurant reviews contain no meaning (neither positive nor negative), are neutral, and simply configure the cognitive component, such as “food”, “restaurant”, “staff”, or “place”, among others. In general, according to the words listed above, customers are satisfied. Furthermore, based on the most-frequent words, it seems that menus offered include steak, chicken, fish, wine and drinks, and tapas.

### 4.1. Cognitive Component

Table 3 shows the spatial distribution of the OTRs at a local level, which are mainly concentrated on two islands (Tenerife and Lanzarote). Table 3 and Figure 3 show strong growth in the number of reviews between 2013 and 2016 and stagnation in 2017. This trend coincides with that observed in a previous work on another mature destination, Attica, Greece [31]. Figure 3 also shows a low seasonality. This may be because the archipelago enjoys a temperate climate throughout the year from the action of the trade winds.

Based on the skewness and kurtosis statistics presented in Table 5, the variable “number of reviews from 2013 to 2017” is not normally distributed. The Kolmogorov-Smirnov test was carried out to confirm the non-normality of all numerical variables related to the number of reviews. The analyses were carried out, taking into account the non-normality, so in mean comparison analyses, a robust ANOVA (Welch and Brown-Forsyth tests) was computed. SPSS 20 (IBM, Armonk, NY, USA) was used in all analyses.

Table 6 shows the top 20 establishments that serve food in the Canary Islands, by location and type of food, with the highest number of reviews, from 2013 to 2017. Regarding the spatial dimension of the image, these 20 restaurants are concentrated on only three islands: Tenerife, Fuerteventura, and Lanzarote. Surprisingly, Gran Canaria, the co-capital of the autonomous community, does not appear. Tenerife included nine of the top 20 restaurants and Lanzarote, six (the most popular). Both the first- and second-ranked establishments have twice as many reviews as the third. Thus, the opinions’ spatial distribution does not reflect the different territories of the Canary Islands at a local community level. It might be that Hard Rock Cafe, an emblematic worldwide chain of restaurants, attracts more reviews because tourists want to share their experience. That is, it generates a high expectation regardless of tourists knowing what they will find before eating there, and even knowing that it is not a restaurant offering local food.

As expected, Spanish, European and Mediterranean gastronomy restaurants are the most numerous (Table 7). However, only two of the host countries and regions are included in the list of the 20 most popular establishments by number of OTRs (Table 6). Although Spain enjoys a great reputation in the field of gastronomy [16], because it has great chefs [74] and high-quality restaurants [14,17], the local Spanish cuisine does not stand out in the list. It is also unusual to observe that fast-food restaurants, which do not reflect the local community’s identity, are significant in the standings, despite the pejorative connotation of such establishments [75]. Behind these numbers, it can be seen that the Canary Islands as a destination has focused on sun, sand, and nature leaving aside gastronomy. This focus results in tourists looking for any kind of restaurant and food but not local, mainly due to the ignorance of what is traditional in the destination.

### 4.2. Appraisive Component

Regarding the evaluative dimension emanating from restaurant OTRs, Table 8 shows that, in general, most reviewers have given an excellent (5*) or very good (4*) scores. This is significant for destination marketing or management organisations (DMO). It seems that visitors to the Canary Islands are satisfied or very satisfied with the destination’s restaurants.

Among the most popular restaurants (counting more than 1300 OTRs), the highest rated (greater than 9 score) are Asian and are located in Tenerife. Restaurants specialising in Spanish local cuisine have a good score but lower than the aforementioned Asian restaurants. The worst rated (6.15) is a British cuisine restaurant in Fuerteventura.

Table 9 shows that an average 82% of total reviews are scored 4* and 5*. As pointed out previously, tourists posting restaurant OTRs are, in general satisfied, or highly satisfied. It also shows other statistics of the variables percentage of reviews out of the total restaurant reviews per score (see the Appendix). Table A1 shows the percentage of reviews out of the total restaurant reviews per score (5*–1*) and per island (Gomera and la Graciosa islands were not included in the analyses due to their low number of restaurants). Table A2 shows a comparison of scores (1–10), means per region and per country.

Table A3 shows the percentage of reviews out of the total restaurant reviews per score (5*–1*) and per region, and Table A4 per country. Results of these tables are relevant for observing differences between scores among the most-frequently reviewed specialty restaurants, and what proportions of reviews give better and worst scores.

In relation to the sentiment analysis, among the 11 most-frequent keywords listed in Table 4, six indicate a positive polarity (good, great, friendly, lovely, excellent, and nice). In the half-million OTRs analysed, more than two million keywords denote positive moods and feelings (Table 10), which represent more than 5% of the total words (including stop words), and the more than 150,000 keywords that express positive recommendations. More than 83% of the ratings qualify restaurants with an excellent or very good score. The average score is higher than 81%. The negative polarity of sentiments, recommendations, and ratings only represents about 10% of positive polarity. Again, tourists consuming food in Canary Islands’ restaurants are satisfied, even though they ignore, or do not prioritize, local food consumption.

## 5. Conclusions

This study aimed to assess the gastronomic image identity related to the construction of the TDI and improving the strategic positioning of the destination. In this sense, user-generated OTRs show not only the image and impressions that tourists perceived in their experience but also the image and identity that will be transmitted to other users. The archipelago offers diverse cuisine, where Oriental restaurants, for example, have better ratings than Spanish local restaurants. These results indicate new trends and preferences at the cultural gastronomic level, as the greatest popularity and appreciation of international cuisine compared to local cuisine, which may have negative effects in socio-cultural sustainability of local communities. Given the analysis of the most frequent affective keywords, one can conclude that tourists of the Canary Islands conveyed a positive image of the destination and had a positive gastronomic experience. The fact that opinions on restaurants include words with positive emotional content can also relate to the high valuations (more than 83% of the opinions about restaurants have scores of excellent and very good). Therefore, the importance of gastronomy as an attraction factor is highlighted.

At an appraisal level, the adjectives used by tourists visiting restaurants show that customers consider a visit to a restaurant as a moment of enjoyment in which relish the experience. In addition, considering that the average valuation of restaurants in the Canary archipelago is above eight points and is maintained throughout the period analysed, one can deduce that this is a destination that offers high quality cuisine in relation to tourists’ expectations.

Referring to the management implications, these results may lead to the conclusion that the Canary Islands as a tourist destination “is doing it well” in satisfying gastronomic tourists, but “is not doing it well” in terms of being a community which offers food that is representative of the local sociocultural identity to visitors. Furthermore, results are in line with [9], who found a notable lack of awareness of regional culinary products among tourists in another Spanish region. In this respect, some kind of strategy should be implemented at the cultural level to improve the values of the local cuisine and create signs of cultural identity based on gastronomy. The development of the local cuisine based on local products as a tourist resource helps the sustainability and identity of the territory, increasing the level of local production and distribution and ensuring respect for tradition and heritage. If tourism sustainable development is attached to the enhancement of local culture and identity, the gastronomic image of the destination should certainly be improved in this respect. Moreover, OTRs generated by users represent a source of valuable information for both restaurants and DMOs. However, the positive effect of eWoM on gastronomy from OTRs shows restaurateurs and managers of tourist destinations that adequate information is provided about restaurants. Using social media to further increase the number of ratings and reviews is an incentive for the tourists to get involved, get better positioning, and improve the image of the destination. Therefore, it is advised that focus be put on the web to further promote gastronomy and restaurants, featuring the experiential component involved and highlighting the local cuisine as a friendly environmental product or health and proximity food.

It should also be noted that the conceptual framework and proposed method allowed us to analyse the gastronomic image identity perceived by tourists transmitted through OTRs to other users, as well as their cognitive and appraisive aspects, especially highlighting the evaluative and affective character of the dining experience. The proposed metrics allow us to compare two or more destinations or types of food delimited in space and time, which may be of use for local communities to know where they are in terms of local gastronomy projection and direct their strategies. The big data (hundreds of thousands of opinions) neutralise the subjectivity of the perceived image and allow for deducing the gastronomic image as a whole. In terms of statistical analyses, relations between the number of reviews per score, percentage of reviews out of total restaurant reviews per score, and per restaurant specialisation (restaurant country and region classifications) are found and validated.

The main limitations of this study include that it was conducted for a specific geographic area, used only TripAdvisor, and the OTRs were not segmented by tourist nationality, which would have opened the possibility for analysing further cultural aspects in studying the impact of dining reviews on the image identity of the destination. Regarding differentiation between locals’ reviews and actual tourists’ reviews, we have assumed that locals’ reviews are written in Spanish. Segmenting by nationality would also help in differentiating tourists’ reviews from locals’ reviews. Another limitation is that restaurants could be classified by more than one type of food category, and in a few cases they seem incompatible; for example, a popular cafe is classified as both Spanish and Thai.

Future research should be deeper and go beyond statistical analysis to analyse the significant relationships between types of food, islands in the archipelago, ratings, and frequent words. A more thorough comparison between island communities should be made, considering the number of tourists and restaurants. This method should be applied to other tourist destinations as well, and in particular, other Spanish regions. Part of this analysis could be done to compare TripAdvisor with other restaurant review platforms, and compositional analysis could allow, for example, comparisons of (graphically and statistically) type shared by type of food in different destinations. Future studies should also be undertaken to understand why the rich Spanish and regional gastronomic cultural background is not the most prominent, popular, and best valued among tourists’ opinions and comments, and to see how local destinations can advance to ensure gastronomic cultural sustainability.

## Figures and Tables

**Table 1:** The Canary Islands as a gastronomic tourist destination.

Metrics	2013	2014	2015	2016	2017
Number of tourists (thousands)	12,038	12,898	12,181	13,005	15,975
Average daily expenditure (€)	125.59	127.48	133.6	135.94	140.18
Average stay (days)	9.6	9.3	9.5	9.4	9.17
Turnover by tourist (€)	1075	1070	1125	1141	1155
Total turnover (mill. €)	12,983	13,901	13,854	14,957	18,450
Average expenditure by restaurants (€)	-	82.17	153.13	148.33	161
Number of restaurants	7480	7435	7541	7617	7666

Source: Authors from TURIDATA (Canary Islands government tourist information system) https://turismodeislascanarias.com/.

**Table 2:** Payment methods and places for tourists’ meals (%) during 2017.

Meal Plan	Tenerife	Lanzarote	Gran Canaria	Fuerteventura	La Palma
Full board at hotels	4.6	3.2	4.3	5.6	3.3
Half board at hotels	20.6	18.4	18.7	18.0	19.5
All-inclusive trip	25.5	34.4	29.3	53.7	18.5
Meals paid in restaurants	54.1	63.4	58.9	51.5	74.1

Source: Authors from Canarian Institute of Statistics (ISTAC).

**Table 3:** Restaurant reviews of the Canary Islands by island and year.

Year	Ten	Lan	GrC	Fue	LaP	Gom	Hie	iGr	% Year
2013	14,485	15,144	5809	6144	156	186	6	4	8.39
2014	22,733	22,154	9099	9541	305	299	16	6	12.83
2015	37,212	32,953	14,776	15,315	619	545	46	13	20.30
2016	55,983	43,336	23,166	21,227	818	847	67	21	29.09
2017	55,171	43,078	26,147	20,631	1034	835	56	17	29.39
% Island	37.12	31.33	15.80	14.57	0.59	0.54	0.04	0.01	

Note: Ten: Tenerife; GrC: Gran Canaria; Lan: Lanzarote; Fue: Fuerteventura; LaP: La Palma; Gom: La Gomera; Hie: El Hierro; and Gra: La Graciosa. Source: Random sample of 500,000 TripAdvisor OTRs.

**Table 4:** Most frequent keywords. Count and percent out of total words.

Rank	Keyword	Count	%	Rank	Keyword	Count	%
1	food	461,423	1.068	26	fantastic	59,929	0.139
2	good	324,818	0.752	27	eat	59,543	0.138
3	great	272,511	0.631	28	little	58,794	0.136
4	restaurant	217,142	0.503	29	cooked	58,172	0.135
5	service	211,716	0.490	30	visited	57,655	0.133
6	staff	202,052	0.468	31	value	56,794	0.131
7	place	160,898	0.372	32	fish	56,759	0.131
8	friendly	151,083	0.350	33	table	56,217	0.130
9	lovely	140,560	0.325	34	like	56,062	0.130
10	excellent	130,190	0.301	35	times	55,457	0.128
11	nice	128,802	0.298	36	wine	54,896	0.127
12	meal	119,243	0.276	37	evening	54,771	0.127
13	best	109,985	0.255	38	drinks	53,964	0.125
14	visit	103,178	0.239	39	holiday	53,709	0.124
15	really	97,854	0.226	40	worth	52,308	0.121
16	just	93,368	0.216	41	definitely	49,826	0.115
17	menu	86,974	0.201	42	atmosphere	49,741	0.115
18	time	84,280	0.195	43	lunch	49,022	0.113
19	recommend	73,968	0.171	44	quality	48,593	0.112
20	night	72,951	0.169	45	came	48,497	0.112
21	went	71,096	0.165	46	chicken	48,232	0.112
22	amazing	65,958	0.153	47	tasty	46,497	0.108
23	steak	65,295	0.151	48	tapas	46,158	0.107
24	delicious	61,338	0.142	49	ordered	45,798	0.106
25	bar	60,987	0.141	50	fresh	43,996	0.102

Note: Total words (including stop words): 43,204,749; unique words: 109,603.

**Table 5:** Number of restaurants per island (*N* and %); and reviews between 2013 and 2017.

Island	Frequency (*N*)	%	Min.	Max.	Mean	Median	Std. Dev	Skew.	Kurt.
Ten	3414	45.2	1	4241	54.36	10	147.28	10.86	223.60
Lan	1129	14.9	1	1932	138.76	40	224.42	3.01	11.95
GrC	1937	25.6	1	1264	40.78	8	90.82	5.36	43.06
Fue	685	9.1	1	4092	106.36	24	251.82	7.91	100.25
LaP	219	2.9	1	139	13.39	6	18.75	3.13	13.02
Gom	134	1.8	1	230	20.24	7	31.72	3.31	15.32
Hie	35	0.5	1	25	5.46	3	5.90	1.69	2.51
iGr	5	0.1	2	32	12.20	9	11.49	1.81	3.79
Total	7558	100			66.16	12	162.77	8.12	131.79

Note: Abbreviations. Min.: minimum number of reviews per restaurant; Max.: maximum; Std. Dev: standard deviation; Skew.: skewness; and Kurt.: kurtosis.

**Table 6:** Top 20 Canary Islands establishments serving food by number of online travel reviews.

Name, Place	Island	Count	Type
Hard Rock Cafe Tenerife, Playa de las Americas	Ten	4241	American, Bar, European
Fado Rock Steak House, Caleta de Fuste	Fue	4092	Italian, American, Steakhouse
Bianco Restaurant, Playa de las Americas	Ten	2010	Italian, Pizza, Mediterranean
Cafe La Ola, Puerto Del Carmen	Lan	1932	International, Spanish, Thai
Waikiki Beach Club, Corralejo	Fue	1873	Italian, Bar, Seafood
Harriet’s Tea Room and Restaurant, Costa Adeje	Ten	1871	Cafe, British, Vegetarian Friendly
Aberdeen Steakhouse, Caleta de Fuste	Fue	1598	European, British, Vegetarian Friendly
15, Caleta de Fuste	Fue	1553	Mediterranean, European, Spanish
Oriental World, Puerto del Carmen	Lan	1542	Chinese, Japanese, Sushi, Asian, Thai, Vegan options
El Maestro, Costa Teguise	Lan	1533	Mediterranean, European, Spanish
El Toro Bravo, Corralejo	Fue	1518	Steakhouse, Mediterranean, European
La Hacienda, Costa Teguise	Lan	1496	Mexican, Spanish, International
Chill Out, Los Cristianos	Ten	1443	Italian, Seafood, Mediterranean
Imperial Tai-Pan, Playa de las Americas	Ten	1363	Chinese, Japanese, Sushi
La Torre Del Mirador, Costa Adeje	Ten	1362	Seafood, International, Mediterranean
Thai Botanico, Playa de las Americas	Ten	1355	Asian, Thai, Vegetarian Friendly
Bombay Babu Torviscas, Costa Adeje	Ten	1337	Indian, Asian, Balti
Friends Lounge Bar & Restaurant, Playa de las Americas	Ten	1296	Barbecue, Mediterranean, Spanish
Cantina Don Rafael, Puerto del Carmen	Lan	1278	Mexican, Mediterranean, Vegetarian Friendly
Ice House, Playa Blanca	Lan	1272	British, Pub, Vegetarian Friendly

Source: Random sample of 500,000 TripAdvisor OTRs.

**Table 7:** More frequent specialisation of establishments serving food in the Canary Islands.

Classification	Specialisation	*N*	%
Region	Mediterranean	2679	35.4
	European	2083	27.6
	International	578	7.6
	Asian	327	4.3
	American	113	1.5
Country	Spanish	2463	32.6
	Italian	907	12.0
	British	343	4.5
	Chinese	251	3.3
	Mexican	101	1.3
Structure	Bar	819	10.8
	Café	566	7.5
	Pizza	566	7.5
	Pub	375	5.0
Kind of food	Vegetarian/Vegan options	740	9.8
	Seafood	615	8.1
	Steakhouse	222	2.9
	Barbeque	153	2.0
	Fast food	151	1.9

Note: a restaurant can be classified by different concepts.

**Table 8:** Reviewer’s scores for the top 20 Canary Islands establishments serving food.

Name, Place	5*	4*	3*	2*	1*	Score
Hard Rock Cafe Tenerife, Playa de las Americas	2235	1107	544	201	154	7.99
Fado Rock Steak House, Caleta de Fuste	2919	768	256	87	62	8.91
Bianco Restaurant, Playa de las Americas	1281	438	187	61	43	8.55
Cafe La Ola, Puerto Del Carmen	1071	470	222	83	86	8.05
Waikiki Beach Club, Corralejo	699	576	318	138	142	7.07
Harriet’s Tea Room and Restaurant, Costa Adeje	1309	406	104	35	17	8.95
Aberdeen Steakhouse, Caleta de Fuste	500	419	261	151	267	6.15
15, Caleta de Fuste	1133	251	109	35	25	8.92
Oriental World, Puerto del Carmen	1048	355	89	27	23	8.86
El Maestro, Costa Teguise	888	389	144	72	40	8.28
El Toro Bravo, Corralejo	825	335	173	89	96	7.81
La Hacienda, Costa Teguise	790	393	153	88	72	7.91
Chill Out, Los Cristianos	1042	260	88	23	30	8.92
Imperial Tai-Pan, Playa de las Americas	1028	219	65	22	29	9.03
La Torre Del Mirador, Costa Adeje	790	341	143	49	39	8.29
Thai Botanico, Playa de las Americas	987	263	67	23	15	9.03
Bombay Babu Torviscas, Costa Adeje	1052	188	56	19	22	9.17
Friends Lounge Bar & Restaurant, Playa de las Americas	896	267	72	27	34	8.79
Cantina Don Rafael, Puerto del Carmen	698	326	148	63	43	8.08
Ice House, Playa Blanca	995	162	51	34	30	9.04

Note: * = bubble. Source: Random sample of 500,000 TripAdvisor OTRs.

**Table 9:** Descriptive statistics of % of reviews out of total restaurant reviews per score (5*–1*).

5*		4*		3*		2*		1*	
Mean	57.32	Mean	25.71	Mean	8.10	Mean	3.67	Mean	5.19
Std. Dev	29.75	Std. Dev	23.95	Std. Dev	14.54	Std. Dev	9.22	Std. Dev	13.01
Skew.	−0.37	Skew.	1.46	Skew.	3.94	Skew.	6.42	Skew.	4.97
Kurt.	−0.62	Kurt.	2.42	Kurt.	19.78	Kurt.	55.69	Kurt.	29.91

Note: * = bubble. Source: Random sample of 500,000 TripAdvisor OTRs.

**Table 10:** Summary of sentiment analysis.

Unit	Feeling+	Feeling−	Recom+	Recom−	5* + 4*	2* + 1*	Score
Number	2,277,646	291,143	151,259	9226	418,037	41,631	61,657.46
Percent	5.2684	0.6734	0.3499	0.0213	83.6074	8.3262	81.4713

Note: + = positive; − = negative; * = bubble. Source: Random sample of 500,000 TripAdvisor OTRs.

## Appendix A

**Table A1:** Percentage of reviews out of total restaurant reviews per score (5*–1*) and island (Brown–Forsythe and Welch tests).

**Island**	**5***			**4***			**3***			**2***			**1***		
	**Mean**		**57.41**	**Mean**		**25.66**	**Mean**		**8.07**	**Mean**		**3.67**	**Mean**		**5.19**
	Standard Dev		29.69	Standard Dev		23.88	Standard Dev		14.43	Standard Dev		9.21	Standard Dev		13.01
	Skewness		−0.38	Skewness		1.47	Skewness		3.95	Skewness		6.45	Skewness		4.98
	Kurtosis		−0.62	Kurtosis		2.45	Kurtosis		19.98	Kurtosis		56.07	Kurtosis		29.98
	Mean	Median	Std. Dev	Mean	Median	Std. Dev	Mean	Median	Std. Dev	Mean	Median	Std. Dev	Mean	Median	Std. Dev
Ten	58.84	60.70	30.30	25.39	22.22	24.78	7.62	2.12	14.91	3.33	0.00	8.96	4.81	0.00	12.56
Lan	60.88	63.64	24.61	23.93	22.73	18.50	7.06	5.00	10.16	3.51	1.79	5.95	4.61	1.43	9.87
GrC	53.88	54.29	31.09	26.27	22.66	25.14	8.84	2.94	15.32	4.27	0.00	10.53	6.73	0.00	16.09
Fue	60.62	62.50	27.08	23.55	22.34	20.72	7.79	5.08	12.12	3.76	0.69	9.84	4.27	0.81	9.99
LaP	44.74	40.00	30.18	36.20	33.33	26.89	11.81	5.77	19.07	3.52	0.00	10.86	3.74	0.00	10.09
Gom	46.87	46.76	31.86	31.91	28.72	26.55	11.79	6.74	19.18	4.58	0.00	10.99	4.85	0.00	13.97
Brown–Forsythe	**			**			**			*			**		
Welch	**			**			**			*			**		

Note: *N* = 7518 (restaurants located in Hierro and La Graciosa islands have not been considered); ** *p*-Value < 0.01; * *p*-Value < 0.05.

As shown in Table A1, between 45% and 61% (mean) of total restaurant reviews are ranked as 5*. Restaurants in Lanzarote and Fuerteventura have the highest percentage of reviews ranked 5*, and restaurants located in La Palma have the lowest percentage of 5* reviews (44.74%). Restaurants in Gran Canaria have the highest percentage of 1* reviews (6.73%). Restaurants located in La Palma and Gomera have the highest percentage of 4* and 3* reviews. Median differences in the percentage of ranked reviews vary along islands (*p*-Values lower than 0.01 and 0.05).

**Table A2:** Comparison of scores (1–10) means per region and per country (Brown-Forsythe and Welch tests). *N* = 7558.

Region		Mean	Median	Std. Dev	Brown-Forsythe Welch	Region		Mean	Median	Std. Dev	Brown-Forsythe Welch.
Mediterranean	No	8.12	8.56	1.91	** **	Spanish	No	8.16	8.53	1.75	
	Yes	8.23	8.42	1.21			Yes	8.15	8.40	1.57	
European	No	8.13	8.52	1.86	** **	Italian	No	8.13	8.47	1.71	** **
	Yes	8.24	8.42	1.14			Yes	8.34	8.61	1.57	
International	No	8.15	8.50	1.69		British	No	8.15	8.48	1.72	** **
	Yes	8.24	8.57	1.64			Yes	8.43	8.62	1.09	
Asian	No	8.15	8.50	1.71		Chinese	No	8.17	8.50	1.69	** **
	Yes	8.24	8.48	1.23			Yes	7.74	8.06	1.76	
American	No	8.17	8.50	1.68	* *	Mexican	No	8.17	8.50	1.69	** **
	Yes	7.66	8.13	2.20			Yes	7.43	7.71	1.71	

Note: Descriptive of variable score (1–10): Mean 8.16; Standard Deviation 1.69; Skewness −1.89; Kurtosis 5.51; ** *p*-Value < 0.01; * *p*-Value < 0.05.

In Table A2, there are statistically significant differences between scores of Mediterranean restaurants (mean 8.23) and those not classified as Mediterranean (8.12). There are also differences between scores of European and American restaurants. Restaurants classified as American have a lower mean score (7.66) than restaurants not-classified as American. There are no significant differences regarding scores obtained by Spanish and non-Spanish restaurants. However, there are differences in mean scores for Italian and non-Italian restaurants, British and non-British, Chinese and non-Chinese, and Mexican and non-Mexican restaurants. For instance, restaurants classified as Mexican and Chinese got lower scores, and restaurants classified as British and Italian got higher scores.

**Table A3:** Percent of reviews out of total restaurant reviews per score (5*–1*) and per region (Brown-Forsythe and Welch tests). *N* = 7558.

Region		5*				4*				3*				2*				1*			
		Mean	Med	Std. Dev	B-F W	Mean	Med	Std. Dev	B-F W	Mean	Med	Std. Dev	B-F W	Mean	Med	Std. Dev	B-F W	Mean	Med	Std. Dev	B-F W
Mediterranean	No	56.60	60.00	33.33	** **	26.07	20.83	27.69	* *	8.28	0.00	17.01		3.52	0.00	10.51	* *	5.52	0.00	15.15	** **
	Yes	58.64	59.94	21.72		25.06	24.19	14.84		7.78	6.27	8.34		3.94	2.19	6.19		4.59	1.85	7.66	
European	No	56.63	59.87	32.63	** **	26.21	21.74	26.92	** **	8.19	0.00	16.55		3.58	0.00	10.42		5.39	0.00	14.67	** **
	Yes	59.15	60.00	20.21		24.41	23.92	13.19		7.87	6.67	8.91		3.91	2.59	4.77		4.66	2.50	6.92	
International	No	57.03	59.34	29.78	** **	26.02	22.92	24.08	** **	8.10	3.45	14.66		3.66	0.00	9.23		5.18	0.00	13.11	
	Yes	60.82	64.29	29.19		22.06	20.00	21.88		8.09	4.59	13.01		3.77	0.00	9.08		5.27	0.00	11.66	
Asian	No	57.19	59.80	30.08	* *	25.84	22.73	24.29	** **	8.09	3.23	14.76		3.68	0.00	9.38		5.19	0.00	13.17	
	Yes	60.22	61.22	20.09		22.89	21.70	14.21		8.24	6.46	8.29		3.49	2.26	4.20		5.16	2.74	8.82	
American	No	57.37	60.00	29.69		25.75	22.70	23.96		8.12	3.57	14.60		3.64	0.00	9.11		5.12	0.00	12.92	** **
	Yes	54.16	54.43	33.24		23.11	20.86	23.22		7.20	2.70	9.86		5.91	0.00	14.49		9.61	1.35	17.45	

Note: ** *p*-Value < 0.01; * *p*-Value < 0.05; B-F: Brown-Forsythe test; W: Welch test.

In Table A3, international restaurants have 60.82% of total reviews ranked as 5*, which compared to non-international restaurants is a significant difference. On the other hand, it is worth noting also that 9.61% of total reviews of American restaurants are ranked as 1*, and there is a significant difference with those non-classified as American.

**Table A4:** Percent of reviews out of total restaurant reviews per score (5*–1*) and per country (Brown–Forsythe and Welch tests). *N* = 7558.

Country		5*				4*				3*				2*				1*			
		Mean	Med	Std. Dev	B-F W	Mean	Med	Std. Dev	B-F W	Mean	Med	Std. Dev	B-F W	Mean	Med	Std. Dev	B-F W	Mean	Med	Std. Dev	B-F W
Spanish	No	57.97	60.26	30.41	** **	24.93	21.74	24.32	** **	8.15	3.23	15.18		3.55	0.00	9.08		5.41	0.00	13.75	* *
	Yes	55.99	57.14	28.31		27.33	25.00	23.07		8.00	4.17	13.14		3.93	0.00	9.49		4.73	0.00	11.32	
Italian	No	56.70	59.00	29.99	** **	26.07	23.08	24.29	** **	8.29	3.49	15.11	** **	3.72	0.00	9.36		5.22	0.00	13.09	
	Yes	61.89	64.53	27.52		23.11	21.14	21.12		6.67	3.74	9.30		3.34	0.00	8.11		4.98	0.00	12.39	
British	No	57.05	59.56	30.09	** **	25.87	22.70	24.32	** **	8.14	3.26	14.74		3.69	0.00	9.34		5.24	0.00	13.24	** **
	Yes	63.09	64.63	20.62		22.30	22.50	13.46		7.35	5.41	9.64		3.21	2.15	6.17		4.06	2.23	6.20	
Chinese	No	57.53	60.00	29.87	** **	25.68	22.50	24.12		8.03	3.33	14.57	* *	3.65	0.00	9.29		5.11	0.00	12.92	* *
	Yes	51.21	52.00	25.19		26.69	25.38	18.26		10.26	7.14	13.51		4.26	2.40	6.34		7.59	2.70	15.24	
Mexican	No	57.45	60.00	29.78	** **	25.69	22.62	23.97		8.08	3.45	14.57		3.64	0.00	9.19	* *	5.13	0.00	12.98	** **
	Yes	47.76	49.41	26.20		27.16	24.58	22.03		9.33	8.33	12.17		5.95	3.90	10.99		9.79	5.14	14.24	

Note: ** *p*-Value < 0.01; * *p*-Value < 0.05; B-F: Brown-Forsythe test; W: Welch test.

As can be seen from Table A4, Chinese and Mexican restaurants have higher percentages of 1* ranked reviews, and lower percentages of 5* ranked reviews. On the other hand, British restaurants have the highest percentage of 5* ranked reviews (63.09% of total reviews). These differences are statistically significant.
